# Uniqueness of codes using semidefinite programming

**DOI:** 10.1007/s10623-018-0589-8

**Published:** 2018-11-30

**Authors:** Andries E. Brouwer, Sven C. Polak

**Affiliations:** 1 Amsterdam, The Netherlands; 2 Korteweg-De Vries Institute for Mathematics, University of Amsterdam, Amsterdam, The Netherlands

**Keywords:** Code, Binary code, Uniqueness, Semidefinite programming, Golay, 94B99, 05B30

## Abstract

For $n,d,w \in \mathbb {N}$, let *A*(*n*, *d*, *w*) denote the maximum size of a binary code of word length *n*, minimum distance *d* and constant weight *w*. Schrijver recently showed using semidefinite programming that $A(23,8,11)=1288$, and the second author that $A(22,8,11)=672$ and $A(22,8,10)=616$. Here we show uniqueness of the codes achieving these bounds. Let *A*(*n*, *d*) denote the maximum size of a binary code of word length *n* and minimum distance *d*. Gijswijt et al. showed that $A(20,8)=256$. We show that there are several nonisomorphic codes achieving this bound, and classify all such codes with all distances divisible by 4.

## Introduction

Let $\mathbb {F}_2:=\{0,1\}$ denote the field of two elements and fix $n \in \mathbb {N}$. A *word* is an element $v \in \mathbb {F}_2^n$. For two words $u,v \in \mathbb {F}_2^n$, their *(Hamming) distance* $d_H(u,v)$ is the number of *i* with $u_i \ne v_i$. A (binary) *code* is a subset of $\mathbb {F}_2^n$. For any code *C*, the *minimum distance* $d_{\text {min}}(C)$ ($\in \mathbb {R}\cup \{\infty \}$) of *C* is the minimum distance between any pair of distinct code words in *C*. The *weight*$\text {wt}(v)$ of a word $v \in \mathbb {F}_2^n$ is the number of nonzero entries of *v*. A (binary) *constant weight code* is a binary code in which all code words have a fixed weight *w*. Then *A*(*n*, *d*, *w*) is defined as the maximum size of a binary constant weight *w* code of minimum distance at least *d*. Moreover, *A*(*n*, *d*) is the maximum size of a binary code of minimum distance at least *d*. A binary constant weight *w* code $C\subseteq \mathbb {F}_2^n$ with $d_{\text {min}}(C)\ge d$ is called an (*n*, *d*, *w*)-code. A binary code $C\subseteq \mathbb {F}_2^n$ and $d_{\text {min}}(C) \ge d$ is called an (*n*, *d*)-code.

Using semidefinite programming, some upper bounds on *A*(*n*, *d*, *w*) have recently been obtained that are equal to the best known lower bounds: it has been established that $A(23,8,11)=1288$ (see [15]), and that $A(22,8,11)=672$ and $A(22,8,10)=616$ (see [14]). We show using the output of the corresponding semidefinite programs that the codes of maximum size are unique (up to coordinate permutations) for these *n*, *d*, *w*.

For unrestricted (non-constant weight) binary codes, the bound $A(n,d)=A(20,8)\le 256$ was obtained in [7], implying that the quadruply shortened extended binary Golay code of size 256 is optimal. The quadruply shortened extended binary Golay code is a linear $(n,d)=(20,8)$-code of size 256 and has all distances divisible by 4.

Up to equivalence there are unique $(24-i,8)$-codes of size $2^{12-i}$ for $i=0,1,2,3$, namely the *i* times shortened extended binary Golay codes [2]. However, it turns out that the 4 times shortened extended binary Golay code is not the only (20, 8)-code of size 256. We classify such codes with all distances divisible by 4, and find 15 such codes.

## The semidefinite programming upper bound

Following [7, 14, 15], we start by describing semidefinite programming upper bounds on *A*(*n*, *d*) and *A*(*n*, *d*, *w*). Fix $n,d,w\in \mathbb {N}$ and let *N* be either $\mathbb {F}_2^n$ or the set of words in $\mathbb {F}_2^n$ of weight *w*. For $k \in \mathbb {Z}_{\ge 0}$, let $\mathcal {C}_k$ be the collection of codes $C \subseteq N$ with $|C|\le k$. We define

$$\begin{aligned} \mathcal {C}_k(D) := \{C \in \mathcal {C}_k \,\, | \,\, C \supseteq D, \, |D|+2|C{\setminus } D| \le k \}, \,\, \text { for}~D \subseteq N. \end{aligned}$$

Note that then $|C \cup C'|= |C| + |C'| -|C\cap C'| \le 2|D| + |C {\setminus } D| + |C' {\setminus } D| -|D| \le k$ for all $C,C' \subseteq \mathcal {C}_k(D)$. Furthermore, for any function $x : \mathcal {C}_k \rightarrow \mathbb {R}$ and $D \in \mathcal {C}_k$ we define the $\mathcal {C}_k(D) \times \mathcal {C}_k(D)$-matrix $M_{k,D}(x)$ by $M_{k,D}(x)_{C,C'} : = x(C \cup C')$.

1 $$\begin{aligned} A_k(n,d,w):=&\max \left\{ \sum _{v \in N} x(\{v\})\,\, |\,\,x:\mathcal {C}_k \rightarrow \mathbb {R}_{\ge 0}, \,\, x(\emptyset )=1, x(S)=0 \text { if}~d_{\text {min}}(S)<d,\right. \nonumber \\&\quad \qquad \left. \,\, M_{k,D}(x) \succeq 0 \text { for each}~D\ \text {in}~\mathcal {C}_k\right\} . \end{aligned}$$

(Here $X \succeq 0$ means: *X* positive semidefinite.) Then $A_k(n,d,w)$ is an upper bound on *A*(*n*, *d*, *w*). Similarly, one obtains an upper bound $A_k(n,d)$ on *A*(*n*, *d*) by setting $N:=\mathbb {F}_2^n$ in (1), so that $\mathcal {C}_k$ is the collection of unrestricted (not necessarily constant weight) codes of size at most *k*. It can be proved that $A_2(n,d)$ and $A_2(n,d,w)$ are equal to the classical Delsarte linear programming bound in the Hamming and Johnson schemes respectively [5].

Let *G* be the set of distance preserving permutations of *N*. In case of constant weight-codes, $G=S_n$, where $S_n$ denotes the symmetric group on *n* elements, but if $n=2w$ the group *G* is twice as large, since then taking complements is also a distance preserving permutation of *N*. In case of non-constant weight codes, $G= S_2^n \rtimes S_n$, where $S_2^n$ denotes the direct product of *n* copies of $S_2$. Let $\Omega _k$ be the set of *G*-orbits of non-empty codes in $C_k$ and let $\Omega _k^d \subseteq \Omega _k$ be those orbits that correspond to codes with minimum distance at least *d*. By averaging an optimum *x* over all $x\circ g$ for $g\in G$, one obtains the existence of a *G*-invariant optimum solution to (1). Here $\circ$ denotes (function) composition, so $x \circ g (C)= x(g(C))$ for $C \in \mathcal {C}_k$.

The original problem is equivalent to the much smaller problem in which the constraint is added that *x* is *G*-invariant. We will write $y_{\omega }$ for the common value of a *G*-invariant function *x* on codes *C* in orbit $\omega$. Hence, the matrices $M_{k,D}(x)$ become matrices $M_{k,D}(y)$ and we have considerably reduced the number of variables in (1). Moreover, a block diagonalization $M_{k,D}(y) \mapsto U_{k,D}^T M_{k,D}(y) U_{k,D}$ can be obtained reducing the sizes of the matrices involved to make the computations in (1) tractable (see [14, 15] for the reductions, where we note that the reductions used in [14] are obtained by an adaptation of the method of [8]).

It can be seen (cf. [7, 14]) that the nonnegativity condition on *x*, and hence on *y*, is already imposed by positive semidefiniteness of all matrices $M_{k,D}(x)$. When solving the semidefinite program with a computer, we add the constraints $y_{\omega } \ge 0$ seperately, by adding $1\times 1$ blocks $(y_{\omega })$ which are required to be positive semidefinite. This will allow us to easily determine which variables $y_{{\omega }}$ will be necessarily zero in any optimum solution. This may yield necessary conditions on all optimal codes, which may give uniqueness or a classification of the optimal codes.

### Information about maximum size codes

Suppose that we have an instance of *n*, *d* or *n*, *d*, *w* for which $A_k(n,d)=A(n,d)$ or $A_k(n,d,w)=A(n,d,w)$, respectively. We want to obtain information about codes attaining these bounds from the semidefinite programming output. The semidefinite program (1) can be written as follows:

2 $$\begin{aligned} A_k(n,d,w) = \max \left\{ \sum _{\omega \in \Omega _k^d } b_{\omega } y_{\omega }\,\,\big |\,\, M= F_{\emptyset } - \sum _{\omega \in \Omega _k^d } F_{\omega } y_{\omega } \succeq 0 \right\} . \end{aligned}$$

Here $b_{\omega _0}=|N|$, where ${\omega _0} \in \Omega _k^d$ corresponds to the orbit of a code of size 1 in $\mathcal {C}_k$, and $b_{\omega } =0$ for all other $\omega \in \Omega _k^d$. Moreover, *M* is a (large) block diagonal matrix that consists of blocks $U_{k,D}^TM_{k,D}(y)U_{k,D}$ (which are reduced versions of the blocks $M_{k,D}(y)$ that are required to be positive semidefinite in (1)) and blocks $(y_{\omega })$. The matrix $F_{\omega }$ is a matrix of the same size as *M* with entries the coefficients of $-y_{\omega }$ in the corresponding entries of *M*. For each orbit $\omega$, the matrix $F_{\emptyset }$ is a matrix of the same size as *M* with entries the constant coefficients in the corresponding entries of *M*. For two real-valued square matrices *A*, *B* of the same size, we write $\langle A, B\rangle := \text {tr}(AB^T)$. The dual program of (2) then reads

3 $$\begin{aligned} \min \left\{ \langle F_{\emptyset }, X \rangle \,\,\big |\,\, \langle F_{\omega }, X\rangle = b_{\omega } \text { for all}~\omega \in \Omega _k^d ,\,~X\succeq 0 \right\} . \end{aligned}$$

If (*M*, *y*) is any optimum solution for (2) and *X* is an optimum solution for (3) with the same value, then $\langle M, X\rangle =0$. As $M \succeq 0$ and $X\succeq 0$, we have in particular $y_{\omega } X_{\omega }=0$ for the separate $1 \times 1$-blocks $(y_{\omega })$ in *M* and $(X_{\omega })$ in *X*, where $(X_{\omega })$ denotes the $1\times 1$-block in *X* corresponding to the $1\times 1$-block $(y_{\omega })$ in *M*. Thus

$$\begin{aligned} X_{\omega }>0\ \text {in any optimum solution to}~(2) \,\,\, \Longrightarrow \,\, y_{\omega }=0\ \text {in all optimum solutions to}~(3). \end{aligned}$$

If $y_{\omega }=0$ for all solutions to (1) with objective value $A_k(n,d,w)=A(n,d,w)$, then for any code *C* of maximum size there is no subcode $D\subset C$ with $D \in \omega$. (Suppose otherwise, then one constructs a feasible solution to (1) by putting $x(S)=1$ for $S \in C_k$ with $S \subseteq C$ and $x(S)=0$ else, and hence by averaging over *G* there exists a feasible *G*-invariant solution with $y_{\omega } >0$, a contradiction.) So orbit $\omega$ does not appear in any code of maximum size. Hence we know which orbits $\omega \in \Omega _k^d$ cannot occur in a code of maximum size. We will call these orbits *forbidden* orbits.

We used the solver SDPA-GMP [11, 16] to conclude which orbits are forbidden. The semidefinite programming solver does not produce exact solutions, but approximations up to a certain precision. In our case the approximations are precise enough to verify (with certainty) that certain orbits are forbidden. See the Appendix for details.

## Self-orthogonal codes

If $u,v \in \mathbb {F}_2^n$, we define $(u\cap v) \in \mathbb {F}_2^n$ to be the word that has 1 at position *i* if and only if $u_i=v_i=1$. The following equality is well-known and will be used often throughout the paper:

4 $$\begin{aligned} d_H(u,v)= {{\mathrm{wt}}}(u) + {{\mathrm{wt}}}(v) -2 {{\mathrm{wt}}}(u \cap v), \,\,\, \text { for all}~u,v \in \mathbb {F}_2^n. \end{aligned}$$

The function $(u,v) \mapsto {{\mathrm{wt}}}(u \cap v) \pmod {2}$ is a non-degenerate symmetric $\mathbb {F}_2$-bilinear form on $\mathbb {F}_2^n$. If $(u,v)=0$, then *u* and *v* are called *orthogonal*. A code *C* is *self-orthogonal* if $(u,v)=0$ for all $u,v \in C$. Given a code *C*, the *dual code* $C^{\perp }$ is the set of all $v \in \mathbb {F}_2^n$ that are orthogonal to all $u\in C$. A code *C* is called *self-dual* if $C=C^{\perp }$. For small *n*, self-dual codes are classified by Pless and Sloane [13].

## Constant weight codes

With semidefinite programming three exact values of *A*(*n*, *d*, *w*) have been obtained. In [15], it is found that $A_3(23,8,11)=1288$, matching the known lower bound and thereby proving that $A(23,8,11)=1288$. Similarly, in [14], the upper bounds $A(22,8,10)\le 616$ and $A(22,8,11)\le 672$ are obtained, which imply $A(22,8,10)=616$ and $A(22,8,11)=672$. The latter two upper bounds are in fact instances of the bound $B_4(n,d,w)$, which is a bound in between $A_3(n,d,w)$ and $A_4(n,d,w)$.

### Definition 4.1

($B_4(n,d,w)$) The bound $B_4(n,d,w)$ is defined by replacing in the definition of $A_4(n,d,w)$ from (1) the matrix $M_{4,\emptyset }(x)$ by (the much smaller matrix) $M_{2,\emptyset }(x)$. See [14] for details.

In this section we show that the codes attaining these bounds are unique up to coordinate permutations, using the information about forbidden orbits obtained from the semidefinite programming output. In order to prove uniqueness of the (23, 8, 11)-code of maximum size, we start by proving uniqueness of the (24, 8, 12)-code of maximum size. The uniqueness of this code can already be obtained from the classical linear programming bound. Below, and also later, we will need the following definition. The *distance distribution* $(a_i)_{i=0}^n$ of a code $C \subseteq \mathbb {F}_2^n$ is the sequence given by $a_i := |C|^{-1} \cdot |\{(u,v) \in C \times C \,\, | \, \, d_H(u,v)=i\}|$, for $i=0,\ldots ,n$. The computational results we used to conclude uniqueness of the mentioned codes, are stated seperately (in (5), (6), (7) and (8) below) at the beginning of each proof.

### *A*(24, 8, 12)

#### Proposition 4.1

Up to coordinate permutations there is a unique (24, 8, 12)-code of size 2576. An example is given by the set of words of weight 12 in the extended binary Golay code.

#### Proof

Let *C* be a (24, 8, 12)-code of size 2576. The classical linear programming bound in the Johnson scheme (which is equal to $A_2(n,d,w)$) gives maximum 2576. Moreover, one has

5 $$\begin{aligned} a_i =0 \text { for }~i \notin \{0,8,12,16,24\}. \end{aligned}$$

This information can be obtained immediately from the dual solution: the linear program contains constraints $a_i \ge 0$. If the corresponding dual variable is $>0$, then $a_i=0$ in all optimum solutions to the linear program.^1^

Consider the $\mathbb {F}_2$-linear span $F:=\langle C \rangle$ of *C*. Note that *C*, hence *F*, is self-orthogonal, so $|F|\le 2^{24/2}=2^{12}$. Since $|F|\ge |C| >2^{11}$ and *F* is linear, we must have $|F|=2^{12}$, so *F* is self-dual. Let $u \in F$, $u \ne 0$. The sets $\{ u + x \, |\, x \in C\} \subseteq F$ and $C\subseteq F$ have non-empty intersection, because both sets have size $2576> |F|/2$. So $u+x=y$ for some $x,y \in C$. But then $\text {wt}(u) = d_H(x,y) \ge 8$, as *C* has minimum distance 8. It follows that *F* has minimum distance 8, and we conclude that *F* is the extended binary Golay code. So *C* is the set of weight 12 words in the extended binary Golay code. $\square$

### *A*(23, 8, 11)

#### Proposition 4.2

Up to coordinate permutations there is a unique (23, 8, 11)-code of size 1288. An example is given by the set of words of weight 11 in the binary Golay code.

#### Proof

Let *C* be a (23, 8, 11)-code of size 1288. With the solution of the semidefinite program $A_3(23,8,11)$ (which is 1288) from [15] one obtains, by considering the forbidden orbits from the semidefinite programming output:^2^

6 $$\begin{aligned} \text {if}~x,y \in C\ \text {then}~ d_H(x,y) \le 16. \end{aligned}$$

Construct a code *D* of length 24, weight 12 and size 2576 as follows: add a symbol 1 to every codeword of *C*, put it in *D* and put also the complement of the resulting word in *D*. Then *D* has minimum distance 8 by (6). Hence *D* is the set of weight 12 words of the extended Golay code *F* by Proposition 4.1. The automorphism group of the extended binary Golay code acts transitively on the coordinate positions [9]. Hence, *C* is the set of weight 11 words in the binary Golay code. $\square$

### *A*(22, 8, 11)

#### Proposition 4.3

Up to coordinate permutations there is a unique (22, 8, 11)-code of size 672.

#### Proof

Let *C* be a (22, 8, 11)-code of size 672. First one concludes that $a_{14}=0$ using the semidefinite program $B_4(22,8,11)$ from [14]. This is explained in more detail in the appendix: if $a_{14}>0$, then $a_{14} \ge 2/672$ and $a_{10}+a_{14}+a_{18}+a_{22} \ge 318/672$ (by Proposition 5.6 below). We add these two constraints to the program $B_4(22,8,11)$. The resulting bound is strictly smaller than 672, so $a_{14}=0$ in any (22, 8, 11)-code of size 672.

Subsequently, by considering the forbidden orbits in the solution of the semidefinite program $A_3(22,8,11)$ from [15] with the added constraint that $a_{14}=0$ (the solution of $A_3(22,8,11)$ with this added constraint is 672) one obtains:

7 $$\begin{aligned} \text {if}~x,y,z \in C\ \text {then}~d_H(x,y) \in \{0,8,12,16\} \ \text {and}~\text {wt}(x+y+z)\in \{7,11,15\}. \end{aligned}$$

Let *D* be the collection of $672+672 = 1344$ codewords of length 24 of the form 10*x* with $x\in C$ together with their complements, and let $F = \langle D \rangle$ be the $\mathbb {F}_2$-linear span of *D*. All distances in *D* belong to $\{8,12,16\}$ by (7), so *D*, and hence also *F*, is self-orthogonal, which implies $|F|\le 2^{24/2} = 2^{12}$. Since all words in *D* have weight divisible by 4 and *F* is self-orthogonal, all words in *F* also have weight divisible by 4.

The code *F* contains words of forms 01*x*, 10*y*, 11*z* and 00*u*. Each form occurs at least 672 times, so $|F| \ge 4 \cdot 672 > 2^{11}$, hence $|F| = 2^{12}$ and *F* is self-dual.

To show that *F* is the extended binary Golay code, it suffices to prove that all words in *F* have weight $\ge 8$, i.e., that no word in *F* has weight 4. Words of *F* are sums of words 10*x* with $x \in C$, possibly together with the all-ones word. So we must prove that sums of words 10*x* do not have weight 4 or 20. A sum of words 10*x* starts with 00 or 10 and is the sum of an even or odd number of words 10*x*, respectively.

Words in *F* starting with 00 form a subcode $F_{00}$ of *F* of size $2^{10} = 1024$. If $u \in F_{00}$, then $\{u+10y \,|\, y \in C\} \cap \{10x \,|\, x \in C\} \ne \emptyset$, as there are 1024 words in *F* starting with 10 but $|C|=672> 1024/2$. So $u=10x+10y$ for some $x,y \in C$, hence $F_{00} = \{10x + 10y \, | \, x,y \in C\}$. However, distances 4 and 20 do not occur in *C*, so words in $F_{00}$ do not have weight 4 or 20.

The 1024 words in *F* starting with 10 are formed by the coset $10x+ F_{00}$ (with $x \in C$ arbitrary but fixed) and hence are a sum of three elements of the form 10*x* with $x \in C$. But such a sum has weight 8, 12 or 16 by (7), implying that words in *F* starting with 10 do not have weight 4 or 20.

Therefore weights 4 and 20 do not appear in *F*, so *F* is indeed the extended binary Golay code. As the automorphism group of the extended binary Golay code *F* acts 2-transitively on the coordinate positions [9], this implies that *C* is unique. $\square$

### *A*(22, 8, 10)

#### Proposition 4.4

Up to coordinate permutations there is a unique (22, 8, 10)-code of size 616.

#### Proof

Let *C* be a (22, 8, 10)-code of size 616. First one concludes that $a_{14}=0$ using the semidefinite program $B_4(22,8,10)$ from [14]. This is explained in more detail in the appendix: if $a_{14}>0$, then $a_{14} \ge 2/616$ and $a_{10}+a_{14}+a_{18} \ge 208/616$ (by Proposition 5.5 below). We add these two constraints to the program $B_4(22,8,10)$. The resulting bound is strictly smaller than 616, so $a_{14}=0$ in any (22, 8, 10)-code of size 616.

Subsequently, by considering the forbidden orbits in the solution of the semidefinite program $A_3(22,8,10)$ from [15] with the added constraint that $a_{14}=0$ (the solution of $A_3(22,8,10)$ with this added constraint is 616) one obtains:

8 $$\begin{aligned} \text {if}~x,y,z \in C\ \text {then}~d_H(x,y) \in \{0,8,12,16\} \ \text {and}~\text {wt}(x+y+z)\in \{6,10,14,22\}. \end{aligned}$$

Let $F=\langle C \rangle$. Since *C* is self-orthogonal and has words of weights divisible by 2 but not by 4, *F* is self-orthogonal and has half of the weights divisible by 4 and half of the weights divisible by 2 but not by 4. Both halves of *F* have size $\ge |C|=616$, but *F* has size $\le 2048$ as it is self-orthogonal. So $|F|=2048$ and *F* is self-dual.

Let $E\subseteq F$ be the subcode of *F* consisting of all words with weight divisible by 4. For each $u \in E$, we have $C \cap \{u+y \,|\, y \in C\} \ne \emptyset$ (as $|C|=616 >1024/2$), so $u=x+y$ for some $x,y \in C$. Hence $E=\{x+y \, | \, x,y \in C\}$. By (8), no word in *E* has weight 4. So weight 4 does not occur in *F*.

If any word *u* in *F* has weight 2 then it is in $F {\setminus } E = x+ E$ (with $x \in C$ arbitrary). So it is the sum of three words in *C*. But such sums do not have weight 2 by (8), hence no word in *F* has weight 2. So *F* is a self-dual code of minimum distance 6. As the self-dual $(n,d)=(22,6)$-code is unique (cf. [13]), *F* is unique. Hence also *C* is unique, as it is the collection of weight 10 words of *F*. (Note that two weight 10 words in *F* have distance $0 \pmod {4}$, so distance at least 8, since $d_H(u,v)={{\mathrm{wt}}}(u)+{{\mathrm{wt}}}(v)-2{{\mathrm{wt}}}(u \cap v)$ for any two words $u,v \in F$.) $\square$

## Unrestricted (20, 8)-codes of size 256

Recently, Gijswijt et al. [7] proved that $A(20,8)=256$, with the semidefinite program $A_4(n,d)$ from (1). An example of a code attaining this bound is the four times shortened extended binary Golay code, which has distance distribution

9 $$\begin{aligned} a_0=1, \,\, a_8=130,\,\, a_{12}=120, \,\,a_{16}=5,\,\,a_i=0 \ \text {for all other}~i. \end{aligned}$$

This code is formed by the words starting with 0000 in the extended binary Golay code with these first four coordinate positions removed.

Two binary codes $C,D \subseteq \mathbb {F}_2^n$ are *equivalent* if *D* can be obtained from *C* by first permuting the *n* coordinates and by subsequently permuting the alphabet $\{0,1\}$ in each coordinate separately.

Up to equivalence there are unique $(24-i,8)$-codes of size $2^{12-i}$ for $i=0,1,2,3$, namely the *i* times shortened extended binary Golay codes [2]. In this section we show that there exist several nonisomorphic (20, 8)-codes of size 256. First we show that there exist such codes with different distance distributions. Subsequently we classify such codes with all distances divisible by 4.

We start by recovering information about possible distance distributions from the semidefinite program $A_4(20,8)$. Write $\omega _t \in \Omega _k$ for the orbit of two words at Hamming distance *t*. From a code *C* with distance distribution $(a_i)$, one constructs a feasible solution to (1) by putting $x(S)=1$ for $S \in C_k$ with $S \subseteq C$ and $x(S)=0$ else, and hence by averaging over *G* one obtains a feasible *G*-invariant solution with variables $y_{\omega }$. This solution has

$$\begin{aligned} y_{\omega _t}&= \frac{1}{|G|} \sum _{g \in G} x\circ g(\{x,y\}) = \frac{t!(20-t)!}{|G|}|\{(u,v) \in C^2 \,\, : \,\, d_H(u,v)=t \}|\nonumber \\&= \frac{|\{(u,v) \in C^2 \,\, : \,\, d_H(u,v)=t \}|}{2^{20} \left( {\begin{array}{c}20\\ t\end{array}}\right) } = \frac{|C| a_t}{2^{20} \left( {\begin{array}{c}20\\ t\end{array}}\right) }, \end{aligned}$$

where $\{x,y\}$ is any pair of words with distance *t* and $G=S_2^{20} \rtimes S_{20}$. So we can add linear constraints on the $a_i$ as linear constraints on the variables $y_{\omega _t}$ to our semidefinite program.

The inner distribution $(a_i)$ is not determined uniquely by the requirement that it is an optimal solution of the semidefinite program $A_4(20,8)$ from (1).^3^ We find minimum possible values for some of the $a_i$ for the case where all distances are even as follows. For any code *C*, the $a_i$ ($i\ne 0$) are integer multiples of 2 / |*C*|. So for any (20, 8)-code of size 256, if $a_{16}<1$ then $a_{16}\le 254/256$. With the constraint $a_{16} \le 254/256$ the semidefinite program returns an objective value strictly smaller than 256. So $a_{16} \ge 1$. Similarly, we find $a_8 \ge 126$ and $a_{12} \ge 96$. If we simultaneously add the constraints $a_8 \le 126$, $a_{12} \le 96$ and $a_{16} \le 1$, the semidefinite program returns 256 as objective value, and the values of $y_{\omega _{10}}$ and $y_{\omega _{14}}$ force $a_{10}=a_{14}=16$. Therefore, apart from the 4 times shortened extended binary Golay code, also a code with

10 $$\begin{aligned} a_1=1,\,\,a_{8}=126,\,\, a_{10}=16, \,\, a_{12}=96,\,\, a_{14}=16,\,\, a_{16}=1,\,\,a_i=0\ \text {for all other}~i, \end{aligned}$$

is allowed by the program $A_4(20,8)$. Such a code exists, as the following construction demonstrates.

Start with the extended binary Golay code *F* containing the weight 8 word *u* with all 1s in the first eight positions. As $A(24-8,8)=A(16,8)=32$ (see [3]), there can be at most 32 words in *F* starting with 8 zeroes. These form a linear subcode *E* of *F*. As any word in *F* has an even number of 1s at the first eight positions, there are at most $2^7=128$ distinct cosets $E+v$ in *F*. As $32 \cdot 128=2^{12}=|F|$ it follows that $|E|=32$ and there are exactly 128 distinct cosets $E+v$.

So if we specify a string of 8 symbols with an even number of ones and take all words in *F* having these 8 fixed symbols in the first 8 positions, we obtain a subcode *D* of *F* of size 32 and minimum distance at least 8. Choose the following 8 specifications, each giving a subcode *D* of size 32 and minimum distance 8.

$$\begin{aligned} \begin{matrix} 00000000 \\ 11000000 \\ 10100000 \\ 10010000 \\ 10001000 \\ 10000100 \\ 10000010 \\ 10000001 \end{matrix} \quad \text { and then replace the first}~8\ \text {coordinates by }\quad \,\, \begin{matrix} 0000 \\ 1100 \\ 1010 \\ 1001 \\ 0110 \\ 0101 \\ 0011 \\ 1111 \end{matrix}. \end{aligned}$$

This yields a (20, 8)-code of size $8\cdot 32= 256$ in which distances 10 and 14 occur. Note that this code indeed has minimum distance at least 8: first observe that each code *D* has minimum distance at least 8. Then note that for two different specifications the first part (the first 8 positions) had distance at most 2 before the replacement, so the second part has distance at least 6. After the replacement of the first part, the first 4 positions have distance at least 2, so two words obtained from different specifications have, after the replacement, in total distance at least $2+6=8$. One verifies by computer that its distance distribution is given by (10). So, there exist (20, 8)-codes of maximum size with distance distribution (9) as well as with (10).

### Unrestricted (20, 8)-codes of maximum size with all distances divisible by 4

In this section we give a classification of the (20, 8)-codes of size 256 with all distances divisible by 4. An example of such a code is the quadruply shortened extended binary Golay code *B*, which is linear. There is, up to equivalence, only one such code, since the automorphism group of the extended Golay code acts 5-transitively on the coordinate positions [2]. (Moreover, Dodunekov and Encheva [6] have proved that there exists, up to equivalence, only one *linear* (20, 8)-code of size 256.) The shortened extended binary Golay code contains 5 words of weight 16, forming a subcode *D*. Since the minimum distance is 8, each of those words must have the 0’s at different positions. So we can assume that

11 $$\begin{aligned} D=\begin{matrix} 00001111111111111111 \\ 11110000111111111111 \\ 11111111000011111111 \\ 11111111111100001111 \\ 11111111111111110000 \end{matrix}, \end{aligned}$$

with linear span $\langle D \rangle \subseteq B$ of dimension 4. So *B* is a union of 16 cosets $u+\langle D\rangle$. If we replace a coset $u + \langle D \rangle$ by its complement $\mathbf {1}+u + \langle D \rangle$, we obtain another (20, 8)-code of maximum size that is not linear, so this is really a different code. Note that all distances remain divisible by –but not equal to– 4, as $d_H(x,y)\in \{8,12\}$ for any $x \in u + \langle D \rangle$ and $y \in B {\setminus } (u + \langle D \rangle )$, so $d_H(\mathbf {1}+x,y) \in \{8,12\}$. By replacing any of the 16 cosets $u+\langle D \rangle$ in *B* with $\mathbf {1}+u+\langle D \rangle$, we obtain $2^{16}=65536$ codes with all distances divisible by 4.

In this section we will first prove that any maximum-size (20, 8)-code with all distances divisible by 4 is equivalent to one of the $2^{16}$ thus obtained codes. Secondly, we will obtain (by computer) that these $2^{16}$ codes can be partitioned into 15 equivalence classes.

In order to prove the first result, we start by proving two auxiliary propositions. Let *C* be any (20, 8)-code of size 256 with all distances divisible by 4 and containing $\mathbf {0}$, the zero word. Define $E:=\langle C, \mathbf {1} \rangle$ to be the linear span of *C* together with the all-ones vector.

#### Proposition 5.1

Up to a permutation of the coordinate positions, the codes *E* and $\langle B, \mathbf {1}\rangle$ are the same.

#### Proof

After the constraints $a_i=0$ if $4 \not \mid i$ and $a_{20} \ge 2/256$ are added to the ordinary LP-bound for $(n,d)=(20,8)$, the linear program returns a solution strictly smaller than 256. Therefore $a_{20}=0$ in any (20, 8)-code of size 256 with all distances divisible by 4. As $A(20,8,8)=130$ (cf. [1]) and $A(20,8,4)=\lfloor 20/4\rfloor =5$, one has $a_{8}\le 130$ and $a_{16} \le 5$. Moreover, the LP-bound contains the inequalities $a_8-a_{12}-3a_{16}+5 \ge 0$ and $-a_{8} -a_{12} +31 a_{16} +95 \ge 0$ (given that $a_{20}=0$). We add those two equations and use that $a_{16} \le 5$ to obtain that $a_{12}\le 120$. As $256=1+a_8+a_{12}+a_{16}$, the distance distribution of any (20, 8)-code of size 256 with all distances divisible by 4 is given by (9). Moreover, the values in (9) are not mere averages: as $A(20,8,8)=130$ and $A(20,8,4)=5$, the number of words at distance *i* from *any* word $u \in C$ is specified by (9).

Since *C* is self-orthogonal and has all distances divisible by 4, also *E* is self-orthogonal and has all distances divisible by 4. Furthermore, *E* has dimension 9. To see this, note that $\mathbf {1} \notin C$ since $a_{20} = 0$, so  $|E|\ge 257$, so $|E| \ge 512$. On the other hand, $\dim E <10$, as *E* is self-orthogonal with all distances divisible by 4, but there does not exist a self-dual code of length 20 with all distances divisible by 4 (cf. [12]). So $\dim E =9$ and $|E|=512$, implying that

12 $$\begin{aligned} \text {for every word}~u \in E\ \text {one has}~u\in C \ \text {or}~\mathbf {1}+u \in C. \end{aligned}$$

For any code we write $A_i$ for the number of words of weight *i*. Since *C* has weights $A_0=1$, $A_8=130$, $A_{12}=120$, $A_{16}=5$, we conclude that *E* has weights

$$\begin{aligned} A_0=1,\, A_4=5, \,A_8=250,\, A_{12}=250, \,A_{16}=5,\, A_{20}=1. \end{aligned}$$

The orthogonal complement $E^\perp$ of *E* has dimension 11, and is a union $E \cup (a+E) \cup (b+E) \cup (c+E)$. Here *a*, *b*, *c* have even weight (because $\mathbf {1} \in E$), so each of $\langle a,E\rangle$ and $\langle b,E\rangle$ and $\langle c,E \rangle$ is self-dual. This means that *a*, *b*, *c* are mutually non-orthogonal.

Look at Pless [12] to find the self-dual codes of length $n=20$ and dimension 10. There are 16 such codes, but we can forget about those with $A_8 < 250$. There is a unique self-dual code of length $n=20$ and dimension 10 with $A_8 \ge 250$, namely $M_{20}$ with weight enumerator

$$\begin{aligned} A_0 = 1,\, A_4 = 5,\, A_6 = 80,\, A_8 = 250,\, A_{10} = 352, \,A_{12}=250,\, A_{14}=80,\, A_{16}=5,\, A_{20}=1, \end{aligned}$$

and *E* is the subcode of $M_{20}$ consisting of the words of weight divisible by 4, hence is unique. This means that *E* does not depend on the particular choice of *C* (up to a permutation of the coordinates), proving the desired result. $\square$

**Table 1:** The (20, 8)-codes of size 256 with all distances divisible by 4. The quadruply shortened extended binary Golay code $B=C_1$ is the union $\cup _{i=1}^{16} (u_i+ \langle D \rangle )$. The other codes $C_j$ ($j=2,\ldots ,15$) are obtained from *B* by replacing the coset $u_i+\langle D \rangle$ by $\mathbf {1}+u_i + \langle D \rangle$ if there is a 1 in entry $(u_i,C_j)$ in the above table

Coset representative	$C_1$	$C_2$	$C_3$	$C_4$	$C_5$	$C_6$	$C_7$	$C_8$	$C_9$	$C_{10}$	$C_{11}$	$C_{12}$	$C_{13}$	$C_{14}$	$C_{15}$
$u_{1\phantom {0}} = 00000000000000000000$		1	1	1	1	1	1	1	1	1				1	
$u_{2\phantom {0}} = 00000101010101011010$			1	1	1	1	1	1	1	1	1	1	1		1
$u_{3\phantom {0}} = 00001001011001101100$				1	1		1			1	1	1	1	1	
$u_{4\phantom {0}} = 00001100001100110110$					1						1				1
$u_{5\phantom {0}} = 10100000010101101001$						1	1	1	1	1	1	1	1		
$u_{6\phantom {0}} = 10100101000000110011$								1				1		1	1
$u_{7\phantom {0}} = 10101001001100000101$									1				1	1	1
$$u_{8\phantom {0}} = 10101100011001011111$$															
$u_{9\phantom {0}} = 11000000011000110101$										1	1	1	1	1	1
$u_{10} = 11000101001101101111$													1	1	1
$$u_{11} = 11001001000001011001$$															
$$u_{12} = 11001100010100000011$$															
$$u_{13} = 01100000001101011100$$															
$$u_{14} = 01100101011000000110$$															
$$u_{15} = 01101001010100110000$$															
$$u_{16} = 01101100000001101010$$															

#### Proposition 5.2

Let *C* be any (20, 8)-code of size 256 with all distances divisible by 4 containing $\mathbf {0}$. Then *C* is invariant under translations by weight 16 words from *C*.

#### Proof

Clearly, if $a,b,c \in \mathbb {F}_2^{20}$ with ${{\mathrm{wt}}}(a)=16$ and $a+b+c=\mathbf {1}$, then $d_H(b,c) = 4$. Now let $b \in C$ be arbitrary, and $a \in C$ a weight 16 vector. Then we have $a+b+c \ne \mathbf {1}$ for all $c \in C$. So $\mathbf {1}+a+b \notin C$ while $\mathbf {1}+a+b \in \langle C,\mathbf {1} \rangle$. Hence, $a+b \in C$, by (12). So indeed, *C* is invariant under translations by weight 16 words from *C*. (This in particular implies that *C* contains the 4-dimensional linear span of the weight 16 vectors.) $\square$

Fix representatives $u_1,\ldots ,u_{16}$ for the cosets $u_i+\langle D \rangle$ of the linear quadruply shortened binary extended Golay code *B* (see Table 1 for a possible choice). Using Propositions 5.1 and 5.2, we obtain the first main result of this section.

#### Proposition 5.3

Let *C* be any (20, 8)-code of size 256 with all distances divisible by 4. Then *C* is equivalent to *B* with some of the cosets $u_i+\langle D \rangle$ replaced by $\mathbf {1}+u_i+\langle D \rangle$.

#### Proof

By applying a distance preserving permutation to *C*, which is possible by Proposition 5.1, we may assume that *C* contains $\mathbf {0}$ and that $\langle C,\mathbf {1} \rangle = \langle B, \mathbf {1} \rangle$. Then *C* contains 5 weight 16 vectors of the form (11). By Proposition 5.2, the code *C* is a union of 16 cosets $u+\langle D \rangle$, for some vectors *u*. The code $\langle B, \mathbf {1} \rangle =\langle C, \mathbf {1} \rangle$ is a union of cosets $u_i+\langle D \rangle$ together with their complements $\mathbf {1}+u_i + \langle D \rangle$. This implies by (12) that each coset of *C* has the form $u_i+\langle D \rangle$ or $\mathbf {1}+u_i+\langle D \rangle$ (and *C* cannot contain both $u_i$ and $\mathbf {1}+u_i$ at the same time as $a_{20}=0$), as required. $\square$

It remains to classify the $2^{16}=65536$ codes obtained from *B* by replacing some of the cosets $u_i+\langle D \rangle$ by $\mathbf {1}+u_i+\langle D \rangle$. For this we use the graph isomorphism program nauty [10]. For any code *C* of word length *n* containing *m* codewords, a graph with $2n+m$ vertices is created: one vertex for each codeword $u \in C$ and two vertices $0_i$ and $1_i$ for each coordinate position. Each code word *u* has neighbor $0_i$ if $u_i=0$ and $1_i$ if $u_i=1$ ($i=1,\ldots ,n)$. Moreover, there are edges $\{0_i,1_i\}$ ($i=1,\ldots ,n$).

All code words have degree *n* and the coordinate positions have (in this case) larger degree. An automorphism of this graph permutes the codewords and permutes the coordinate positions. In this way one finds a subgroup of $S_2^n \rtimes S_n$ that fixes *C* and the question of code equivalence is transformed into a question of graph isomorphism. With the program nauty we compute a canonical representative for each of the $2^{16}$ mentioned codes. In this way we find that the $2^{16}$ codes from Proposition 5.3 can be partitioned into 15 equivalence classes. See Table 1 for the classification.

#### Proposition 5.4

There are 15 different (20, 8)-codes of size 256 with all distances divisible by 4 up to equivalence. $\square$

## Appendix: Approximate solutions

The semidefinite programming solver does not produce exact solutions, but approximations up to a certain precision. Here we show that we have enough precision to conclude (6)–(8).

Let (*M*, *y*) be feasible for (2) with optimum value at least *A*(*n*, *d*, *w*) and suppose that *X* is an approximation of the dual program. That is, we have $X \succeq 0$^4^ and for all $\omega \in \Omega _k^d$:

13 $$\begin{aligned} \langle X, F_{\omega } \rangle = b_{\omega } + \epsilon _{\omega }, \end{aligned}$$

for small $\epsilon _{\omega }$. Consider one particular $\omega \in \Omega _k^d$. There is a $1 \times 1$-block $(y_{\omega })$ in *M* and hence also a corresponding $1 \times 1$-block $(X_{\omega })$ in *X*. Remove these $1\times 1$ blocks from *M* and *X* and call the resulting matrices $M'$ and $X'$. Then

14 $$\begin{aligned} 0&\le \langle M', X' \rangle = \langle M,X\rangle -y_{\omega }X_{\omega } \nonumber \\&= \langle F_{\emptyset },X\rangle - \sum _{\omega \in \Omega _k^d} y_{\omega }b_{\omega } + \sum _{\omega \in \Omega _k^d} y_{\omega } \epsilon _{\omega } -X_{\omega } y_{\omega }, \end{aligned}$$

as $M'$ and $X'$ are positive semidefinite, where we used (13) and the definition of *M* from (3) in the second equality. Note that $\sum _{\omega \in \Omega _k^d} y_{\omega }b_{\omega }$ is bounded from below by |*C*|, and each $y_{\omega }$ is bounded from above by $y_0$ (this can be done since all $2 \times 2$ principal submatrices in the semidefinite program (2) are positive semidefinite), which is bounded from above by 1. Hence

15 $$\begin{aligned} X_{\omega }y_{\omega } \le \langle F_{\emptyset },X \rangle - |C| + \sum _{\omega \in \Omega _k^d}\epsilon _{\omega }. \end{aligned}$$

The numbers $\epsilon _{\omega }$ are easily calculated from the dual solution, just as the dual approximate objective value $\langle F_{\emptyset },X\rangle$. So we find a constant $c_{\omega }$ from the semidefinite programming dual approximation *X* such that

16 $$\begin{aligned} X_{\omega }y_{\omega } \le c_{\omega }. \end{aligned}$$

In the case of *A*(23, 8, 11) one can conclude with the semidefinite program $A_3(23,8,11)$, which can be solved with SDPA-GMP [11, 16] within minutes, that

17 $$\begin{aligned} y_{\omega } \le 10^{-90} \end{aligned}$$

for all orbits corresponding to codes not satisfying (6). Let $\omega$ be an orbit for which (17) holds. If there exists a code *C* of maximum size containing a subcode $D\subset C$ with $D \in \omega$, then one constructs a feasible solution to (1) by putting $x(S)=1$ for $S \in C_k$ with $S \subseteq C$ and $x(S)=0$ else, and hence by averaging over *G* there exists a feasible *G*-invariant solution with $y_{\omega } \ge 1/|G|$ (this lower bound is not best possible, but sufficient). In our case, $G=S_{23}$, so $y_{\omega } \ge 1/23! > 10^{-23}$, which gives a contradiction with (17). In this way, one verifies that all orbits not satisfying (6) are forbidden, thereby establishing (6). We used a seperate java program to check that $X\succeq 0$ (in fact, $X \succ 0$) and to compute the error terms as in (13) and (15).

Next, we consider the cases $A(22,8,10)=616$ and $A(22,8,11)=672$. First we assume that $a_{14}=0$ for all maximum-size $(22,8,10)=616$ and (22, 8, 11)-codes *C*. We write $\omega _t$ for the orbit of two words at Hamming distance *t*. Adding the constraint $y_{\omega _{14}}=0$ to the programs $A_3(n,d,w)$ for these two cases of *n*, *d*, *w* gives $A_3(n,d,w)=A(n,d,w)$. In this way one shows, in the same way as in the previous paragraph, that all orbits not satisfying (7) and (8) are forbidden, provided that $a_{14}=0$. So in order to establish (7) and (8), it remains to prove that

18 $$\begin{aligned} \text {if}~C\ \text {is a maximum-size}~(22,8,10)\text {- or}~(22,8,11)\text {-code, then}~a_{14}=0. \end{aligned}$$

Suppose to the contrary that *C* is a code as in (18), yet $a_{14}>0$. Then $a_{14} \ge 2/|C|$. We will show that this is not possible by adding constraints to the (large) program $B_4(n,d,w)$. The semidefinite program will then give $\lfloor B_4(n,d,w)\rfloor < A(n,d,w)$ and we will arrive at a contradiction, as $B_4(n,d,w)$ is an upper bound for *A*(*n*, *d*, *w*). To find a better lower bound on some of the $a_i$, we use the following two propositions. We use in both propositions that for two words *u*, *v* in a constant weight *w* code *C*,

19 $$\begin{aligned} d_H(u,v) \equiv 2 \pmod {4} \,\,\,\,\, \Longleftrightarrow \,\,\,\,\, {{\mathrm{wt}}}(u \cap v) \not \equiv w \pmod {2}, \end{aligned}$$

which follows from (4). In the next two propositions we will call ${{\mathrm{wt}}}(u\cap v)$ the *inner product* of *u* and *v*.

### Proposition 5.5

Let *C* be a (22, 8, 10)-code of size 616 with $a_{14} \ge 2 / 616$. Then $a_{10}+a_{14}+a_{18} \ge 208/616$.

### Proof

Suppose that $a_{10}+a_{14}+a_{18} < 208/616$ (note that $a_{22}=0$ as two weight 10 words cannot have distance 22). Then, by (19), there are at most $206/2=103$ pairs of words in *C* with odd inner product. Let $\{b,b'\} \subseteq C$ be such a pair of words. Starting with $b\in C$, and greedily picking vectors $d \in C$ such that the inner product of *d* with the already chosen vectors is even, we end with a self-orthogonal subcode *B* of *C* of size $\ge 616-103=513$. Starting with $b'$, we repeat the same process to end up with a self-orthogonal subcode $B'$ of *C* (containing $b'$) of size $\ge 513$. Furthermore, $D:= B \cap B'$ has $|D| \ge 512$. (To see this, note that $b,b'\notin D$ with odd inner product. Every word $v \in C {\setminus } (D\cup \{b,b'\})$ has odd inner product with some word in *C* so there are at most $103-1=102$ of such words *v*, as $b,b'$ is already a pair with odd inner product.) Write $F:=\langle D \rangle$. Then *F* is self-orthogonal, as *D* is self-orthogonal.

Since $$\langle B \rangle $$ and $$\langle B' \rangle $$ are self-orthogonal codes, they have dimension at most 11. Since $$\langle B \rangle \ne \langle B'\rangle $$, as $$b \notin \langle B'\rangle $$, we have $$\dim F = \dim \langle B \cap B'\rangle \le \dim ( \langle B \rangle \cap \langle B'\rangle ) \le 10$$. On the other hand, we have $$ |F| >512$$ (as $$D \subseteq F$$ and the zero word is contained in *F*). So $$\dim F=10$$. Moreover, *F* has minimum distance 8. To see this, let *u* be any nonzero word in $$F {\setminus } D$$. Then $$D \cap (u +D) \ne \emptyset $$, as both *D* and $$u+D$$ have size 512 and are contained in $$F {\setminus } \{ \mathbf {0}\}$$, a set of size 1023. So *u* is the sum of two words of *D*, and hence has weight at least 8 (as *D* has minimum distance at least 8).

So *F* is a self-orthogonal code of word length 22, dimension 10 and minimum distance 8. Such a code is the twice shortened extended binary Golay code (see [2]), which does not contain words of weight 10. But all words in $D \subseteq F$ have weight 10, a contradiction. $\square$

### Proposition 5.6

Let *C* be an (22, 8, 11)-code of size 672 with $a_{14} \ge 2 / 672$. Then $a_{10}+a_{14}+a_{18}+a_{22} \ge 318/672$.

### Proof

Suppose that $a_{10}+a_{14}+a_{18}+a_{22} < 318/616$. Then, by (19), there are at most $316/2=158$ pairs of words in *C* with even inner product. Let $\{b,b'\} \subseteq C$ be such a pair of words. Starting with $b\in C$, and greedily picking vectors $d \in C$ such that the inner product of *d* with the already chosen vectors is odd, we end with a subcode *B* of *C* of size $\ge 672-158=514$. Now, add an extra symbol 1 to every codeword in *B*, to obtain a self-orthogonal code *D* of length 23. As *D* is self-orthogonal, $\dim \langle D \rangle \le \lfloor 23/2\rfloor =11$.

Starting with $b' \in C$, we repeat the same process to end up with a subcode $B'$ of *C* (containing $b'$) of size $\ge 514$ such that all pairs of words in $B'$ have odd inner product. Add an extra symbol 1 to every code word in $B'$ to obtain a self-orthogonal code $D'$ of length 23, so $\dim \langle D'\rangle \le 11$. Note that $\langle D \rangle \ne \langle D'\rangle$, as $1b' \notin \langle D\rangle$.

Furthermore, $E:= D \cap D'$ has $|E| \ge 513$ and all words start with 1. (To see this, note that $b,b'\notin B \cap B'$ with even inner product. Every word $v \in C {\setminus } ((B \cap B' ) \cup \{b,b'\})$ has even inner product with some word in *C* so there are at most 157 of such words *v*.) Hence $|\langle E\rangle | \ge 2 \cdot 513$, so $\dim \langle E\rangle \ge 11$. But $\langle D\rangle$ and $\langle D'\rangle$ are distinct codes of dimension $\le 11$, so their intersection has dimension $<11$, hence

$$\begin{aligned} \dim \langle E \rangle =\dim \langle D \cap D'\rangle \le \dim (\langle D\rangle \cap \langle D' \rangle ) <11, \end{aligned}$$

a contradiction. $$\square $$

From a code *C* with distance distribution $(a_i)$, one constructs a feasible solution to (1) by putting $x(S)=1$ for $S \in C_k$ with $S \subseteq C$ and $x(S)=0$ else, and hence by averaging over *G* there exists a feasible *G*-invariant solution. This solution has

$$\begin{aligned} y_{\omega _t}&= \frac{1}{|G|} \sum _{g \in G} x\circ g(\{x,y\}) = \frac{(\frac{t}{2})!(w-\frac{t}{2})!(\frac{t}{2}) !(22-w-\frac{t}{2})!}{|G|}|\{(u,v) \in C^2 \,\, : \,\, d_H(u,v)=t \}| \nonumber \\&= \frac{|\{(u,v) \in C^2 \,\, : \,\, d_H(u,v)=t \}|}{\left( {\begin{array}{c}22\\ w\end{array}}\right) \cdot \left( {\begin{array}{c}22-w\\ t/2\end{array}}\right) \left( {\begin{array}{c}w\\ t/2\end{array}}\right) } = \frac{|C| a_t}{\left( {\begin{array}{c}22\\ w\end{array}}\right) \left( {\begin{array}{c}22-w\\ t/2\end{array}}\right) \left( {\begin{array}{c}w\\ t/2\end{array}}\right) } = \frac{y_{\omega _0} a_t}{\left( {\begin{array}{c}22-w\\ t/2\end{array}}\right) \left( {\begin{array}{c}w\\ t/2\end{array}}\right) }, \end{aligned}$$

where $\{x,y\}$ is any pair of constant-weight *w* words with distance *t* and $G=S_{22}$, the symmetric group on 22 elements.

So we can add linear constraints on the $a_i$ as linear constraints on the variables $y_{\omega _t}$ to our semidefinite program. To the program $B_4(22,8,10)$ we add the constraints $a_{14} \ge 2/616$ and $a_{10}+a_{14}+a_{18} \ge 208/616$. To $B_4(22,8,11)$ we add the constraints $a_{14} \ge 2/672$ and $a_{10}+a_{14}+a_{18}+a_{22} \ge 318/672$. Write $B_4^*(n,d,w)$ for the resulting bound after adding these constraints. We find $B_4^*(n,d,w)<A(n,d,w)$ in both cases (which we verified using the dual solution), which is not possible.^5^ This establishes (18) and hence completes the verification of (7) and (8).

The time needed to solve the semidefinite programs $B_4^*(22,8,11)$ and $B_4^*(22,8,10)$ varied from one to three weeks with sufficient precision to conclude that $B_4^*(n,d,w) < A(n,d,w)$ in these two cases (with SDPA-DD). By contrast, the semidefinite programs for $A_3(22,8,10)$ and $A_3(22,8,11)$ can be solved withvery high precision within minutes (with SDPA-GMP).

The computer programs we used to generate input for the SDP-solver can be found in [4]. Also, the input and output files for the SDP solver can be found in this folder, and a java program to inspect the outputs.

## Footnotes

1 We used SDPA-GMP to solve this LP. The approximate dual solution allows us, with a computation similar to the computation in the Appendix in (13)–(17) below, to give a very small $\varepsilon >0$ such that in any optimum solution, $a_i < \varepsilon$ for $i \notin \{0,8,12,16,24\}$. But if $a_i>0$ for some $(n,d,w)=(24,8,12)$-code *C* with $|C|=2576$, then for this code $a_i \ge 2 / 2576$, by definition of the distance distribution of *C*. Since $\varepsilon<10^{-90} < 2/2576$ for $i \notin \{0,8,12,16,24\}$ it follows that $a_i=0$ for any (24, 8, 12)-code *C* of size 2576.

2 Note that the LP does not give this information: the Delsarte bound is 1417, which is not optimal.

3 By contrast, in all constant weight cases considered in this paper, the values of the $y_{\omega _t}$ give the unique distance distribution of the (unique up to coordinate permutations) (*n*, *d*, *w*)-codes of maximum size.
